# Effects of Roasting Temperature and Time on the Chemical Composition of Argan Oil

**DOI:** 10.1155/2018/7683041

**Published:** 2018-06-07

**Authors:** Rahma Belcadi-Haloui, Abderrahmane Zekhnini, Yassine El-Alem, Abdelhakim Hatimi

**Affiliations:** ^1^Laboratory of Plants Biotechnologies, Faculty of Sciences, BP 8016, Agadir 80 000, Morocco; ^2^Laboratory of Aquatic Systems, Faculty of Sciences, BP 8016, Agadir 80 000, Morocco; ^3^Autonomous Establishment of Control and Coordination of Exports, 23 E, Industrial Zone of Tassila, Agadir 80 000, Morocco

## Abstract

This work aimed at assessing the effects of roasting temperature and duration on chemical composition of argan oil. Thus, argan oils extracted from almonds roasted at different temperatures (75-175°C) and times (10-30 min) were analyzed and compared to a control. The physicochemical parameters (acidity, peroxide value, and absorbance at 232, 270 nm) increased slightly and the fatty acid composition did not show significant variation, regardless of roasting temperature and duration. The browning index increased significantly for temperatures greater than or equal to 100°C. The tocopherols content significantly decreased with roasting temperature and time (from 977.9 to 305.2 mg/kg after roasting at 175°C for 10 min). However, fluctuations are noted as a function of temperature. The phospholipids content increased with roasting temperature and time (from 0.198 % to 1.370 % after roasting at 175°C for 30 min). The decrease in the tocopherols content would be due to their thermolability. The increase in phospholipids and tocopherols content could be explained by better extractability. The results obtained make it possible to conclude that a roasting at 125-150°C / 10 min would allow the development of the organoleptic properties of the oil, notably its hazelnut flavour, without compromising its oxidative stability.

## 1. Introduction

Argan oil extracted from the fruits of* Argania spinosa* L. (Sapotaceae) has many nutritional, cosmetic, and therapeutic properties thanks to its richness in unsaturated fatty acids (UFA) and bioactive substances such as tocopherols and phytosterols [1]. The cosmetic oil is extracted by mechanical press from unroasted almonds. As for the edible oil, it is extracted by press or in the traditional way by hand kneading roasted almonds. Roasting is usually carried out on the wood fire by constantly stirring the almonds until browning. It constitutes an important step in the development of the organoleptic characteristics of the oil, in particular the typical hazelnut aroma appreciated by the consumer.

In industry, heating of oilseeds is carried out with the aim of increasing oil extraction yield, reducing seed moisture, and deactivating lipases and lipoxygenases which could induce fatty acids (FA) oxidation [2]. The oxidation could lead to loss of nutritional value and decreased shelf life [3]. The roasting of oilseeds could also improve the stability of vegetable oils by increasing the extraction yield of antioxidant substances [4, 5]. As regards the effect of roasting on the content of tocopherols which represent the major antioxidants of vegetable oils, the data from the literature are inconsistent. Depending on the nature and variety of oilseeds, roasting may lead to a decrease [5–7] or an increase in tocopherols level in oil [8–10]. Since tocopherols are thermosensitive, their final concentration in the oil will depend essentially on the temperature and the duration of the roasting.

In the case of argan oil, the literature does not report studies of the impact of roasting on the chemical composition of the oil, with the exception of the study by Harhar et al. [11] in which only the duration of the roasting was taken into consideration for a constant temperature of 110°C. The objective of this work was to evaluate the concomitant effects of the temperature and the duration of the roasting of the* A. spinosa *almonds on the chemical composition of the oil. To do this, the roasting was carried out by combining temperatures of 75, 100, 125, 150, and 175°C with times of 10, 20, and 30 min. The chemical composition of the oil related to the content of fatty acids, tocopherols, and phospholipids. The physicochemical parameters considered were acidity, peroxide value (PV), absorbance at 232 nm (K232) and 270 nm (K270), and browning index (assessed by absorbance at 420 nm).

## 2. Materials and Methods

### 2.1. Argan Fruits and Reagents

The fruits of argan tree were collected in the forest of Admine located at 15 km in the east of Agadir city (south of Morocco). Tocopherols and fatty acids homologues were purchased from Sigma-Aldrich (St Louis, USA). Hexane, tetrahydrofuran, chloroform, and cyclohexane were obtained from Merck (Darmstadt, Germany). All other chemicals and solvents were of analytical grade.

### 2.2. Preparation of Argan Oil

Argan fruits were depulpated and obtained seeds were crushed manually. Then, almonds were distributed in batches of 300 g, uniformly spread on a stainless steel plate, and placed in an electric convection oven (Binder ED 115, USA) under continuous aeration. Roasting temperatures of 75, 100, 125, 150, and 175°C were applied for duration of 10, 20, or 30 min. After cooling, the almonds were subjected to a mechanical press for cold extraction of the oil (Komet S87G press, Germany). A control sample of oil was obtained from unroasted almonds.

### 2.3. Determination of Physicochemical Parameters

The acid value (AV) and the peroxide value (PV) were determined according to IUPAC 2.201 [12] and IUPAC 2.501 [13], respectively.

The absorbance at 233 and 270 nm was determined using a Varian DMS 80 spectrophotometer after diluting oil in cyclohexane (1/100, v/v).

The color of oils was evaluated as described by Yoshida et al. [8]. Solutions of 5% of oils in chloroform (w/v) were prepared to determine absorbance at 420 nm in a Varian DMS 80 spectrophotometer.

### 2.4. Fatty Acids Analysis

Fatty acid methyl esters (FAME) were prepared according to AOCS method Ce 1K-07 [14]. FAME were analyzed by gas chromatography (Agilent 6890, USA). The apparatus was equipped with a flame ionisation detector and a BPX70 column (60 m length, 0.32 mm internal diameter, and 0.25 *μ*m film; SGE Europe). Helium was used as the carrier gas at a flow rate of 1 ml/min. The temperature of the column oven was 170°C. The temperature of the injector and the detector was 220°C.

### 2.5. Tocopherols Analysis

The analysis of tocopherols was carried out using high performance liquid chromatography (Flexar, Perkinelmer, USA). The apparatus was equipped with a C18 column (250 mm length, 4 mm internal diameter, and 5 *μ*m particle size; Varian Inc.) and a fluorescence detector. The excitation and detection wavelengths were 290 and 330 nm, respectively. The mobile phase consisted of 2:98 tetrahydrofuran/hexane (v/v) mixture and the flow rate was 1 ml/min. Oil samples were diluted in hexane (0.1g/ml) before analysis.

### 2.6. Determination of Phospholipids Content

The phospholipids content was estimated by measuring phosphorous according to AOCS recommended method Ca 12-5515 [15]. The method is based on ashing the oil sample in the presence of zinc oxide. The phosphorus is then determined by a spectrophotometric measurement as a blue phosphomolybdic acid complex (Varian DMS 80 spectrophotometer). The phospholipids content is obtained by multiplying the phosphorus content by 25.

### 2.7. Statistical Analysis

The Statistica 6 software was used for processing data from three replicates (n = 3). Significant differences from the control were determined using the analysis of variance (ANOVA) followed by Newman-Keuls student method.

## 3. Results and Discussion

### 3.1. Physicochemical Parameters

The effect of roasting on the initial physicochemical characteristics of the argan oil is presented in Table 1. The acidity of 0.18 for the control oil increased significantly under the effect of the roasting whatever the temperature and the duration. The maximum values (0.28-0.3) were recorded for the temperature of 175°C. This result is consistent with previous works on pine nuts, colza, and sesame oils [16–18]. The increase in acidity could be explained by the release of FA following the hydrolysis of the triglycerides which constitute the major components of vegetable oils. It could trigger reactions of oxidative degradation of the oil due to the prooxidative action of free FA [19].

The PV was 0.1 meq O_2_/Kg for the control oil. It significantly increased in oils extracted from almonds roasted at 125°C for 30 min, 150°C for 10 min, and at 175°C for 10, 20, and 30 min durations. PV is a parameter commonly used to evaluate the primary oxidation state of vegetable oils during technological treatments and storage. It reflects the content of hydroperoxides formed by oxidation of the UFA. In this regard, previous studies of rapeseed, sesame, and walnut seeds oils reported an increase in the PV of the oil with an increase in the roasting temperature of the seeds [16, 18, 20]. Our results showed that roasting at elevated temperatures resulted in oil oxidation which could affect its stability during storage.

K232 reflects the content of conjugated dienes (CD) produced from the hydroperoxides of the UFA. K270 permits to evaluate the secondary oxidation products resulting from the decomposition of hydroperoxides [3]. In our study, the K232 value of 0.844 for the control oil increased to 0.878, 0.896, and 0.906, respectively, under the 175°C/10 min, 75°C/30 min, and 100°C/10 min conditions and decreased to 0.814, 0.806, and 0.793, respectively, for the couples temperature (°C)/time (min) of 175/30, 100/20, and 75/10. The absorption at 270 nm also showed variations according to the roasting conditions. Overall, it increased from 0.041 to 0.062, respectively, at 150°C/30 min and 75°C/30 min conditions, compared to the control (0.054). Previous studies reported that increasing temperature and time of seed roasting led to an increase in K232 and K270 [18, 21]. The fluctuations of the CD contents recorded in this work would be due to the instability of these compounds which transform into secondary oxidation products. The increase in the K270 value indicates the formation of undesirable oxidation products such as carbonyl compounds. However, the values of acidity, PV, and K270 encountered under the various conditions of our work remained below the limits set by the Moroccan Standard NM 08.5.090 [22] characterizing the “extra virgin” quality of argan oil (acidity < 0.8; PV < 15 meq O_2_/kg, K270 < 0.35).

**Figure 1:**
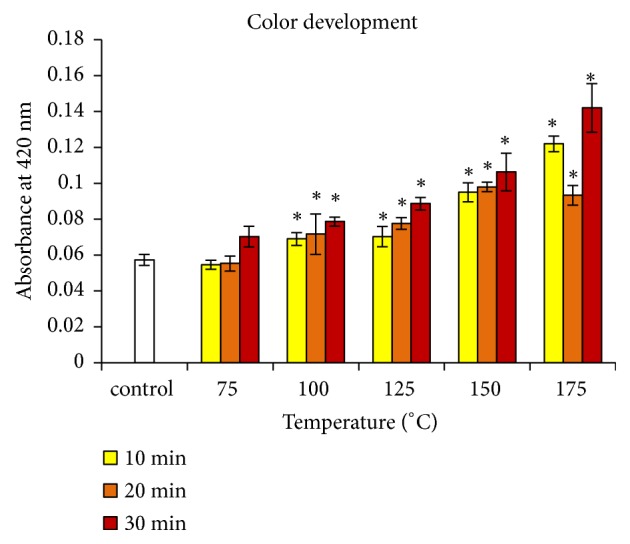
Changes in absorbance (at 420 nm) of argan oil according to roasting temperature and time. ^*∗*^ Significantly different from control (p < 0.05).

The development of the color of argan oil prepared from almonds roasted at different temperatures and times is shown in Figure 1. The absorbance at 420 nm increased significantly from 100°C with both time and temperature. These results are in agreement with data previously reported for other edible oils [9, 10, 17]. They reflect the formation of browning substances from nonenzymatic Maillard-type reactions between reducing sugars and free amino acids or amides [23]. The products of the Maillard reaction are also responsible for the development of the flavour of the edible argan oil. For this reason, roasting constitutes an important step in the edible oil production. Some derivatives of the Maillard reaction also afford numerous food safety benefits [24]. However, others exhibited toxicity [25, 26].

### 3.2. Fatty Acid Composition

The FA composition of oils obtained from unroasted and roasted almonds is shown in Table 2. Argan oil from unroasted almonds consisted of 47.01% oleic and 32.66% linoleic acids as UFA and 13.59% palmitic and 5.49% stearic acids as saturated fatty acids (SFA). These results are consistent with previous works [27, 28]. The roasting did not lead to significant variations of FA composition. Similar results were reported for safflower and rice germ oils [9, 10]. This result would be due to the protective effect of almonds tissues by limiting the contact between UFA and oxygen which is responsible for initiating the oxidative processes [29]. In addition, natural antioxidants such as polyphenols and tocopherols present in almonds tissues could exert a protective effect against UFA oxidation. In general, vegetable oils are estimated by their FA composition. Their nutritional value is related to the richness of essential FA (*ω*3 and *ω*6 families). Argan oil is produced for food and cosmetic purposes. In the process of extracting the edible oil, the roasting of the almonds is systematically carried out for the development of the organoleptic characteristics such as flavour and color. As for the cosmetic oil, it is cold extracted from unroasted almonds. Our results showed that both types of extraction processes result in the same FA profile.

### 3.3. Tocopherols Composition

**Figure 2:**
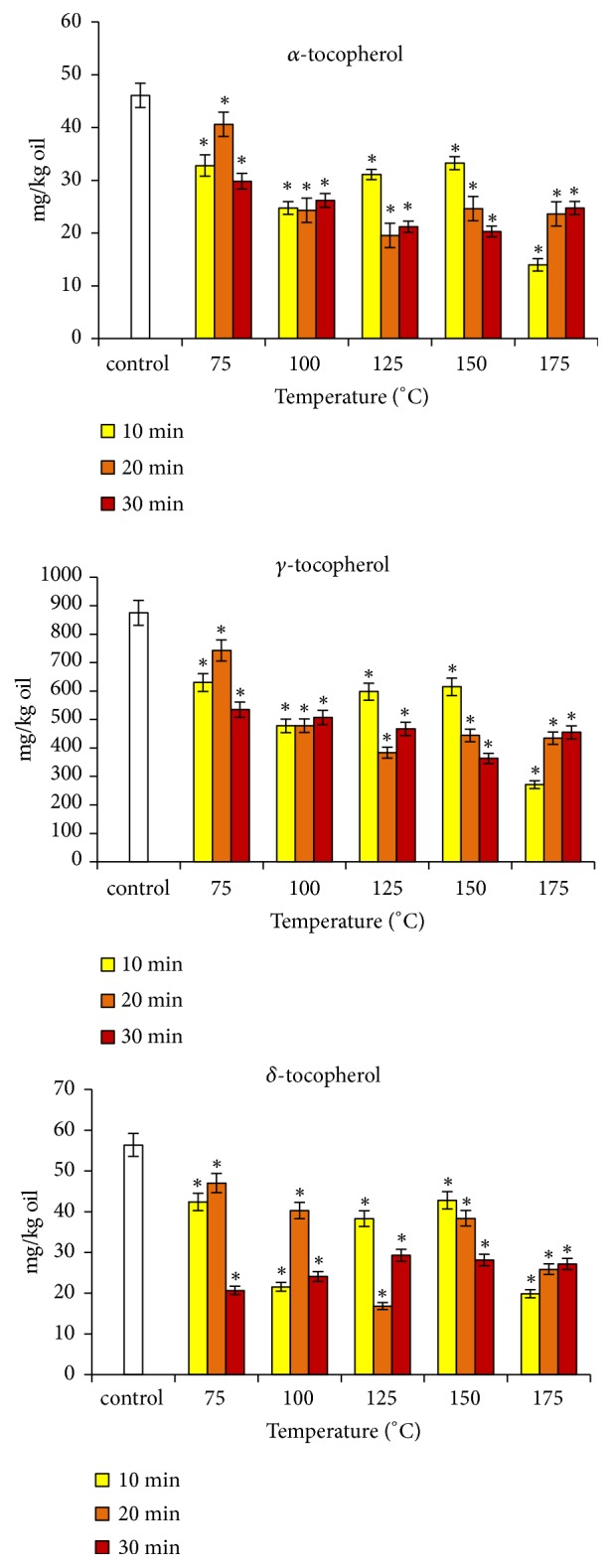
Changes in tocopherols content of argan oil according to roasting temperature and time. ^*∗*^ Significantly different from control (p < 0.05).

The effect of roasting on tocopherols content is shown in Figure 2. The control oil exhibited a total tocopherols level of 977.9 mg/kg with 46.1, 875.5, and 56.3 mg/kg of *α*-, *γ*-, and *δ*-tocopherol. Thus, *γ*-tocopherol represented 89.5% of the total tocopherols of argan oil, *α*-tocopherol 4.7%, and *δ*-tocopherol 5.8%. These results are consistent with data from the literature and confirm the richness of argan oil in *γ*-tocopherol [1]. Roasting caused a significant decrease in tocopherols content. The lowest levels were recorded in the condition 175°C/10 min with values of 32.1, 604.1, and 44.9 mg/kg for *α*-, *γ*-, and *δ*-tocopherol, respectively. The loss was about 69% for the three forms. This showed that the thermal sensitivity is comparable for the three isomers of tocopherols during argan almonds roasting. Our results also exhibited fluctuations of the tocopherols content with increasing temperature and time of roasting. Considering the effect of the roasting time, it appears that the content of the three forms of tocopherols was comparable at the roasting temperature of 100°C. On the other hand, the values were higher for the duration of 10 min when the roasting temperature was 125 and 150°C. Some studies carried out on pumpkin, rapeseed, and sesame seed oils recorded successive decreases and increases in the tocopherols content according to the increase of the roasting temperature and duration [29, 30]. Other works reported either an increase or a decrease in the level of tocopherols as a function of the roasting temperature. As an example, Lee et al. [10] noted an increase in tocopherols content in safflower oil with increasing roasting temperature, and Eitenmiller [31] reported a decrease in content of tocopherols with increasing temperature and roasting time for peanut oil. To explain these variations, two major hypotheses were adopted: (i) the decrease in tocopherols level would be due to their thermolability; (ii) the increase would result from a better extraction due to the destruction of the tocopherol-retaining cellular structures and rupture of their binding to membrane proteins and/or phospholipids [4, 31, 32]. The decrease in the tocopherols level following the almonds roasting constitutes a significant loss of the nutritional value of the edible argan oil, in particular for the high temperatures which are widely used during the artisanal extraction process. In order to preserve the nutritional properties and stability of the argan oil, it would be advisable to avoid high roasting temperatures. This will make it possible to minimize the destruction of tocopherols which are endowed with a high antioxidant power.

### 3.4. Phospholipids Content

**Figure 3:**
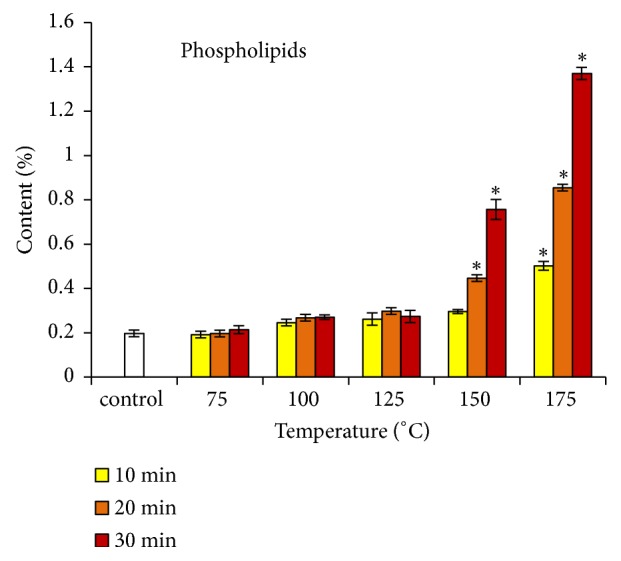
Changes in phospholipids content of argan oil according to roasting temperature and time. ^*∗*^ Significantly different from control (p < 0.05).

The variation of the phospholipids content under the effect of roasting is represented by Figure 3. The control oil showed a content of 0.198%. The values obtained at the roasting temperatures of 75, 100, and 125°C did not differ significantly from that of the control oil. They increased significantly after roasting at temperatures of 150 (20 and 30 min) and 175°C (10, 20, and 30 min). The values also increased with the roasting time (0.447 and 0.756%, respectively, after 20 and 30 min at 150°C, and at 0.503, 0.855, and 1.370%, respectively, after 10, 20, and 30 min at 175°C). These results are in agreement with previous works carried out on safflower, rice germ, argan, pumpkin, and mustard seeds reporting an increase in phospholipids content as a function of temperature and/or duration of roasting [9–11, 33, 34]. As for tocopherols, the increase in the phospholipids level could be explained by a better extractability following the destruction of almonds tissues by roasting.

Several studies have focused on the effects of roasting conditions on the physicochemical characteristics of edible oils. Significant changes in some characteristics such as color, aroma, fatty acid profile, and bioactive compounds have been reported [9, 16]. The heating of oil seeds also leads to the initiation of the oxidation of UFA and the loss of natural antioxidants. The natural antioxidants content strongly influences the oxidation stability of the oil during storage. In this regard, the tocopherols present the most effective antioxidants for reducing lipid oxidation in oils [35]. The *α*-tocopherol also has vitamin activity. Therefore, the roasting process at uncontrolled temperatures could induce a reduction in oxidative stability and a loss of nutritional value of the oil.

The studies on the effects of roasting on the chemical composition and the oxidative stability of argan oil are not numerous. In addition, the temperature used by the authors remains relatively low (110°C) [11, 36, 37] compared to works done on other oilseeds [6, 7, 29, 38], but also with regard to the temperatures applied for the traditional extraction of argan oil intended for commerce or for a familiar use. Indeed, the extraction of argan oil is mainly done using the artisanal process, especially in rural areas of southern Morocco. Almonds from the argan tree are roasted over a wood fire using a stove. They are then ground using a rotary arm grinding stone and the resulting paste is crushed by hand. The addition of small amounts of warm water facilitates the extraction of the oil [39]. The argan oil obtained by this process is reputed to be poorly preserved [40]. Indeed, high roasting temperatures, contact with the metal, and the addition of water are all factors for initiating the oxidation of the oil. In this regard, Mathaüs et al. reported that the traditional oil was characterized by negative sensory attributes after 12 weeks of storage [36]. This result has been attributed in particular to the conditions of manual extraction practiced in the artisanal process. More recently, cooperatives producing argan oil use electric ovens for roasting and the extraction is carried out by press. This allowed an improvement in the quality of the oil compared to the artisanal process [39]. Mathaüs et al. also noted an improvement in the oxidative stability and sensory quality of oil extracted from roasted almonds at 110°C for 30 min [36]. These results were explained by a better extraction of antioxidants and the formation of Maillard reaction products during roasting. In fact, for the same temperature (110°C), other authors reported a significant increase in phospholipids content [11, 37] and a significant decrease in moisture [11]. As for the content of FA and tocopherols, Harhar et al. did not report significant changes [11]. However, the roasting time of 45 min (at 110°C) induced a reduction in the oxidative stability of the oil [11]. This allowed these authors to conclude that 110°C/30 min (temperature/time) represents an optimal roasting condition for preserving the nutritional and the organoleptic properties of argan oil.

In order to better reproduce the roasting conditions used in the two argan oil extraction processes (artisanal and mechanical), we used temperatures between 75 and 175°C for periods of 10 to 30 min. Our results showed that PV and acidity increased with temperature and roasting time with maximum values reaching 175°C/30 min. With regard to tocopherols, the lowest levels were obtained after roasting at 175°C/10 min. The temperatures of 125° and 150°C induced a lower increase in PV and a lower reduction in the tocopherols content especially when the duration of roasting was limited to 10 min. These same conditions caused a significant increase in the browning index. From these results, it can be concluded that heating argan almonds at high temperatures is not recommended, even for short periods. Since the roasting of argan almonds is an important step for the development of the organoleptic characteristics of edible argan oil, particularly its aroma and color, it would be desirable to use temperatures between 125 and 150°C for duration of 10 min. Nevertheless, our study requires additional investigations to evaluate the oxidative stability of oils extracted from roasted almonds at different temperatures and times.

## 4. Conclusion

This study showed that the temperature and the duration of roasting (75 to 175°C, and 10 to 30 min, respectively) had little effect on the physicochemical characteristics of the argan oil. Similarly, the composition of FA was not modified by roasting whatever the temperature and the time used. However, the browning index increased significantly from 100°C, important decrease in the tocopherols content was recorded at 175°C, and significant increase in the phospholipids level was obtained from 150°C/20 min condition. These results make it possible to conclude that roasting at temperatures of 125-150°C for 10 min would allow the development of organoleptic characteristics without compromising the nutritional value and the oxidative stability of the oil.

## Figures and Tables

**Table 1:** Effects of temperature and duration of roasting on the physicochemical characteristics of argan oil.

Roasting conditions	Peroxide value (meq O_2_/Kg)	Acidity (%)	K232	K270
Control	0.10 ± 0.01	0.180 ± 0.020	0.844 ± 0.021	0.054 ± 0.001
75°C				
10 min	0.11± 0.01	0.230 ± 0.010 ^*∗*^	0.793 ± 0.042 ^*∗*^	0.057 ± 0.003^*∗*^
20 min	0.11± 0.01	0.240 ± 0.010 ^*∗*^	0.815 ± 0.014 ^*∗*^	0.054 ± 0.001^*∗*^
30 min	0.11± 0.01	0.240 ± 0.010 ^*∗*^	0.896 ± 0.075 ^*∗*^	0.062 ± 0.002^*∗*^

100°C				
10 min	0.12 ± 0.02	0.207 ± 0.015 ^*∗*^	0.906 ± 0.062 ^*∗*^	0.052 ± 0.001^*∗*^
20 min	0.12 ± 0.01	0.210 ± 0.010 ^*∗*^	0.806 ± 0.097 ^*∗*^	0.048 ± 0.001^*∗*^
30 min	0.12 ± 0.01	0.217 ± 0.015 ^*∗*^	0.846 ± 0.061 ^*∗*^	0.051 ± 0.001^*∗*^

125°C				
10 min	0.11 ± 0.02	0.250 ± 0.010 ^*∗*^	0.858 ± 0.092 ^*∗*^	0.053 ± 0.001^*∗*^
20 min	0.13 ± 0.02	0.240 ± 0.010 ^*∗*^	0.824 ± 0.063 ^*∗*^	0.050 ± 0.001^*∗*^
30 min	0.14 ± 0.01 ^*∗*^	0.220 ± 0.010 ^*∗*^	0.836 ± 0.095 ^*∗*^	0.052 ± 0.003^*∗*^

150°C				
10 min	0.14 ± 0.02 ^*∗*^	0.223 ± 0.012 ^*∗*^	0.860 ± 0.033 ^*∗*^	0.050 ± 0.002 ^*∗*^
20 min	0.13 ± 0.02	0.260 ± 0.017 ^*∗*^	0.834 ± 0.034 ^*∗*^	0.044 ± 0.004 ^*∗*^
30 min	0.12 ± 0.02	0.270 ± 0.010 ^*∗*^	0.824 ± 0.025^*∗*^	0.041 ± 0.003

175°C				
10 min	0.15 ± 0.02 ^*∗*^	0.280 ± 0.010 ^*∗*^	0.878 ± 0.021 ^*∗*^	0.055 ± 0.002 ^*∗*^
20 min	0.15 ± 0.02 ^*∗*^	0.283 ± 0.015 ^*∗*^	0.827 ± 0.031 ^*∗*^	0.053 ± 0.003 ^*∗*^
30 min	0.16 ± 0.02 ^*∗*^	0.300 ± 0.010 ^*∗*^	0.814 ± 0.097^*∗*^	0.052 ± 0.004 ^*∗*^

Values present means ± standard deviations from triplicate measurements.

^*∗*^ Significantly different from control (p < 0.05).

**Table 2:** Effect of temperature and duration of roasting on the content of the argan oil in principal fatty acids.

Roasting conditions	Fatty acid (%)				
	C16:0	C18:0	C18:1	C18:2	C18:3
Control	13.59 ± 0.10	5.49 ± 0.04	47.00 ± 0.05	32.66 ± 0.02	0.085 ± 0.001

75°C					
10 min	12.89 ± 0.09	5.61 ± 0.07	46.79 ± 0.06	33.44 ± 0.04	0.084 ± 0.003
20 min	13.17 ± 0.03	5.44 ± 0.06	47.75 ± 0.05	32.38 ± 0.11	0.082 ± 0.001
30 min	13.17 ± 0.03	5.34 ± 0.05	47.73 ± 0.02	32.50 ± 0.07	0.083 ± 0.002

100°C					
10 min	13.41 ± 0.03	5.46 ± 0.07	46.71 ± 0.08	33.23 ± 0.06	0.090 ± 0.001
20 min	13.50 ± 0.04	5.52 ± 0.05	46.39 ± 0.07	33.30 ± 0.11	0.091 ± 0.001
30 min	13.54 ± 0.02	5.51 ± 0.07	46.21 ± 0.02	33.41 ± 0.06	0.092 ± 0.001

125°C					
10 min	13.48 ± 0.08	5.50 ± 0.05	46.11 ± 0.04	33.61 ± 0.12	0.092 ± 0.001
20 min	13.41 ± 0.03	5.48 ± 0.10	45.88 ± 0.03	33.91 ± 0.06	0.095 ± 0.001
30 min	13.31 ± 0.10	5.52 ± 0.09	46.16 ± 0.17	33.66 ± 0.09	0.100 ± 0.013

150°C					
10 min	13.58 ± 0.01	5.52 ± 0.12	46.08 ± 0.07	33.46 ± 0.03	0.094 ± 0.002
20 min	13.31 ± 0.01	5.36 ± 0.21	46.24 ±0.14	33.72 ± 0.04	0.094 ± 0.004
30 min	13.36 ± 0.01	5.51 ± 0.10	45.82 ± 0.04	33.96 ± 0.01	0.096 ± 0.003

175°C					
10 min	13.33 ± 0.02	5.45 ± 0.10	46.06 ± 0.03	33.80 ± 0.02	0.095 ± 0.002
20 min	13.16 ± 0.02	5.28 ± 0.16	47.16 ± 0.13	33.03 ± 0.03	0.091 ± 0.003
30 min	13.28 ± 0.01	5.47 ± 0.15	47.48 ± 0.41	32.12 ± 0.17	0.086 ± 0.004

Values present mean ± standard deviations from triplicate measurements.

No significant variation was recorded compared to the control.
